# Effect of Periodontal Ligament Stem Cells-Derived Conditioned Medium on Gene Expression and Differentiation of Tumor Necrosis Factor-α-Challenged Osteoblasts

**DOI:** 10.1055/s-0043-1771337

**Published:** 2023-08-10

**Authors:** Poranee Vongsakulpaisarn, Sujiwan Seubbuk Sangkhamanee, Supanee Rassameemasmaung, Hathaitip Sritanaudomchai

**Affiliations:** 1 Department of Oral Medicine and Periodontology, Faculty of Dentistry, Mahidol University, Bangkok, Thailand; 2 Department of Oral Biology, Faculty of Dentistry, Mahidol University, Bangkok, Thailand

**Keywords:** conditioned medium, mesenchymal stem cells, osteoblasts, osteoprotegerin, RANKL, tumor necrosis factor-alpha

## Abstract

**Objectives** Tumor necrosis factor-α (TNF-α) causes bone resorption in periodontitis. It induces the production of receptor activator of NF-κB ligand (RANKL) from osteoblasts, leading to the disturbance of bone homeostasis through RANKL, RANK, and osteoprotegerin (OPG) axis. This study aimed to explore the effect of periodontal ligament stem cells-derived conditioned medium (PDLSCs-CM) on gene expression related to bone homeostasis and the differentiation of TNF-α-challenged osteoblasts.

**Materials and Methods** Human osteoblasts were cultured with 50 ng/mL of TNF-α and 0, 1, 10, and 100 µg/ mL of PDLSCs-CM. Osteoblasts cultured without TNF-α and PDLSCs-CM were served as control. Gene expression of RANKL, OPG, and interleukin-1β (IL-1β) was evaluated by reverse transcription quantitative polymerase chain reaction at 48 hours. The early-stage and late-stage differentiation of TNF-α-challenged osteoblasts without or with PDLSCs-CM was explored by alkaline phosphatase (ALP) activity and alizarin red staining, respectively, at day 1, 3, 6, 9, and 12.

**Statistical Analysis** Mann–Whitney U test was used to analyze the differences in gene expression of TNF-α-challenged osteoblasts at 24 and 48 hours, and Kruskal–Wallis test was used to analyze the effect of PDLSCs-CM on gene expression and ALP activity among all experimental groups using SPSS software version 21.0. Statistical significance was considered with *p* -value less than 0.05.

**Results** Expression of RANKL, OPG and IL-1β was significantly upregulated in TNF-α-challenged osteoblasts compared to the untreated control. The PDLSCs-CM at 1 and 10 μg/mL downregulated gene expression of TNF-α-challenged osteoblasts compared to the group without PDLSCs-CM, but the difference did not reach statistical significance. The ALP activity was decreased in TNF-α-challenged osteoblasts. The addition of PDLSCs-CM did not alter ALP activity of TNF-α-challenged osteoblasts. Alizarin red staining was comparable in the TNF-α-challenged osteoblasts cultured without or with PDLSCs-CM.

**Conclusions** The PDLSCs-CM did not alter gene expression involved in bone homeostasis and differentiation of TNF-α-challenged osteoblasts.

## Introduction

Periodontitis is one of the chronic inflammatory diseases that affects quality of life. Pathophysiology of this disease is caused by microbial challenge stimulating the host immunoinflammatory response. Innate and adaptive immunity are stimulated to release proinflammatory cytokines such as tumor necrosis factor-α (TNF-α) and interleukin-1β (IL-1β). These cytokines can upregulate other inflammatory mediators associated with bone destruction including TNF-α, IL-1β, IL-6, and prostaglandin E2. 1

TNF-α can stimulate osteoblasts to produce receptor activator of NF-κB ligand (RANKL). When RANKL binds to its receptor, RANK, on osteoclast and preosteoclast cell surfaces, it promotes osteoclast recruitment and stimulates osteoclast proliferation and differentiation. 2 This process is inhibited by osteoprotegerin (OPG) that acts as a decoy receptor by binding to RANKL and blocking its interaction with RANK, and OPG is produced by a variety of cell types including osteoblasts. 3 In addition, TNF-α can either activate or inhibit osteoblastic differentiation. TNF-α upregulated ALP activity of osteoblasts in a dose-dependent manner. In contrast, other studies showed that ALP activity was increased by low concentrations but decreased at high concentrations of TNF-α. 2 Thus, osteoblast lineage cells may be an important therapeutic target in the prevention of alveolar bone loss through the modulation of the RANKL/RANK/OPG axis.

Progenitor cells from bone and gingival connective tissue did not provide new connective tissue attachment. 4 5 Instead, healing was characterized mainly by root resorption and ankylosis. 4 On the other hand, periodontal ligament cells can differentiate into cementum-forming cells, bone-forming cells, or fibroblasts; therefore, they possess the ability to reestablish connective tissue attachment with new cementum formation. 6

Periodontal ligament stem cells (PDLSCs) are the mesenchymal stem cells (MSCs) derived from periodontal ligament. They show the ability to regenerate periodontal tissue through the formation of cementum/PDL-like structure and bone 7 and promote adhesion of collagen fibers with newly formed cementum-like structures, mimicking physiological attachment of Sharpey's fibers in an animal study. 8 Transient paracrine actions from PDLSCs are strongly associated with tissue regeneration and wound healing. 9 In addition, PDLSCs possess the ability to suppress immune reactions. 10 However, there are some limitations associated with the use of PDLSCs in tissue regeneration including the risk of tumorigenesis, donor quality, and immune rejection. 11

Research on the use of conditioned medium (CM) of MSCs is growing. The PDLSCs-derived conditioned medium (PDLSCs-CM) contains various growth factors, proinflammatory and anti-inflammatory cytokines, and tissue regenerative agents 11 secreted through either the autocrine or paracrine actions. 12 The advantages of CM are the ease of manufacturing and transportation and no need of donor-recipient matching. 11 Recent studies found that PDLSCs-CM could reduce TNF-α and IL-1β gene expression in lipopolysaccharide-challenged THP-1 cells (monocytoid human cell line) and MO3.13 (oligodendrocyte progenitor cells), as well as IL-1β-challenged chondrocytes, synoviocytes, and meniscus. 13 14 Due to the role of TNF-α in alveolar bone resorption, we aimed to evaluate whether PDLSCs-CM could alter the expression of genes related to bone homeostasis and differentiation of TNF-α-challenged osteoblasts.

## Materials and Methods

### Cell Culture

The PDLSCs obtained from the previous study 15 were cultured in Dulbecco's modified Eagle's medium (DMEM: HyClone, Fisher Scientific, Loughborough, UK) containing 10% fetal bovine serum (FBS: Biochrome, Berlin, DE) and 1% penicillin-streptomycin antimicrobial agent (Gibco, Thermo Fisher Scientific, Loughborough, UK) at 37 °C and 5% CO _2_ . The culture medium was changed every other day. Cells were subcultured after 80 to 90% confluence using 0.25% trypsin/ethylenediaminetetraacetic acid (Gibco, Grand Island, New York, US). The PDLSCs at passage 5-8 were used in this study.

Human osteoblastic cell line, human fetal osteoblastic (hFOB) 1.19, was purchased from American Type Culture Collection (ATCC, Manassas, Virginia, US). According to the manufacturer's instruction, the cells were cultured in a 1:1 mixture of Ham's F12 Medium and Dulbecco's Modified Eagle's Medium without phenol red supplement with 2.5 mM L-glutamine (Gibco, Grand Island, New York, US), 10% FBS, and 0.3 mg/mL G418 (Gibco, Grand Island, New York, US). The hFOBs were seeded in 75 cm ^2^ cell culture flasks (Thermo Fisher Scientific, Waltham, Massachusetts, US) under the standard conditions of 34 °C and 5% CO _2_ . The culture medium was changed every 2 to 3 days.

### Determination of Gene Expression in TNF-α-Challenged Osteoblasts

Osteoblasts (2 × 10 ^5^ cells) were seeded in 6-well plates at least 24 hours to ensure proper attachment. After that, cells were cultured with fresh DMEM mixed with 50 ng/mL TNF-α (R&D Systems, Minneapolis, Minnesota, US) and incubated at 37°C and 5% CO _2_ for 24 and 48 hours. Cells cultured in fresh DMEM without TNF-α were served as control.

Expression of RANKL, OPG, and IL-1β was analyzed by quantitative reverse transcription polymerase chain reaction (RT-qPCR). Briefly, total RNA was extracted using TRIzol reagent (Invitrogen, Carlsbad, California, US) according to the manufacturer's instruction. Purity and concentration of RNA were assessed using nanophotometer (Thermo Fisher Scientific, Waltham, Massachusettes, US). To eliminate any contaminated DNA, DNase I, RNase-free (Thermo Fisher Scientific, Waltham, Massachusettes, US) was used. The purified RNA was reversed transcribed to cDNA using an iScript reverse transcription supermix for RT-qPCR (Bio-Rad, Hercules, California, US) according to the manufacture's instruction. Quantitative PCR was performed to compare the expression of the interested genes using Luna Universal qPCR Master Mix (Luna, Ipswich, Massachusetts, US). Comparative cycle threshold (C _T_ ) was analyzed for relative gene expression with 2 ^-ΔΔCT^ method. Glyceraldehyde 3-phosphate dehydrogenase (GAPDH) was used as internal control gene. The primer sequences for RANKL, OPG, and IL-1β used in this study were shown in Table 1 .

**Table 1:** Primer sequences for RT-qPCR

Genes	Sequences	Product length (bps)	Annealing temperature (°C)	Ref.
RANKL	F: 5′-TGATTCATGTAGGAGAATTAAA CAGG-3′ R: 5′-GATGTGCTGTGATCCAACGA-3′	82	59	Zheng et al 2018 16
OPG	F: 5′-TGAGGAGGCATTCTTCAGGT-3′, R: 5′-CGCTGTTTTCACAGAGGTCA-3′	236	60	Yeom et al 2021 17
IL-1β	F:5′-TGAGGATGACTTGTTCTTTGAAG-3′ R: 5′-GTGGTGGTCGGAGATTCG-3′	115	60	Ballerini et al 2017 14
GAPDH	F: 5′-CTCATTTCCTGGTATGACACC-3′ R: 5′-CTTCCTCCTGTGCTCTTGCT-3′	122	60	Eslaminejad et al, 2010 18

Abbreviations: bps, base pairs; IL-1β, interleukin-1 β; OPG, osteoprotegerin; RANKL, receptor activator of NF-κB ligand; RT-qPCR, reverse transcription-quantitative polymerase chain reaction.

### Preparation of CM from Periodontal Ligament Stem Cells

The PDLSCs were cultured in 75 cm ^2^ cell culture flasks to 80 to 90% confluence. They were washed twice with 10 mL of phosphate buffer saline (PBS) and refreshed with 10 mL of serum-free DMEM. Culture supernatant was collected after 48 hours of incubation and then centrifuged (1000 g, 5 min at 4°C) and filtered through a 0.2 μm syringe filter (Pall corporation, Port Washington, New York, US) to remove cell debris. The CM was concentrated using ultrafiltration with a cutoff of 10 kDa (Invitrogen, Carlsbad, California, US) at 5000 g for 40 minutes and stored at −80°C until used.

### Determination of Protein Concentration in the CM

Protein concentration in PDLSCs-CM was determined by Bradford assay. Briefly, protein standards were prepared using bovine serum albumin (Merck, Darmstadt, DE). The protein standards and unknown samples were added into each well and mixed with 200 μL of the Bradford reagent (Bio-Rad, Hercules, California, US) and incubated at room temperature for 5 to 10 minutes. The measurement of the absorbance was performed at a wavelength of 595 nm. Protein concentration of the unknown samples was determined by calculating the absorbance at 595 nm against the standard curve.

### Determination of Gene Expression of TNF-α-Challenged Osteoblasts Cultured with PDLSCs-Derived CM

The experiment was assigned into five groups as follows:

(1) Osteoblasts cultured in DMEM supplemented with 5% FBS (control group),
(2) Osteoblasts cultured in DMEM with 50 ng/mL TNF-α,
(3) Osteoblasts cultured in 50 ng/mL TNF-α and 1 μg/mL PDLSCs-CM,
(4) Osteoblasts cultured in 50 ng/mL TNF-α and 10 μg/mL PDLSCs-CM,
(5) Osteoblasts cultured in 50 ng/mL TNF-α and 100 μg/mL PDLSCs-CM.

Osteoblasts (2 × 10 ^5^ cells) were seeded in 6-well plates for at least 24 hours. Then, fresh medium was added as assigned and incubated at 37°C and 5% CO _2_ for 48 hours. Determination of gene expression in each group was performed as previously described.

### Determination of Osteoblastic Differentiation through Alkaline Phosphatase Activity and Alizarin Red Staining

Osteoblasts (1 × 10 ^4^ cells) seeded in 96-well plates were cultured in the assigned medium and incubated at 37°C and 5% CO _2_ . Alkaline phosphatase (ALP) activity and alizarin red staining were evaluated at 1, 3, 6, 9, and 12 days.

For ALP activity, the medium was removed. The cells were washed three times with PBS. Two hundred microliters of ALP assay buffer (Ab171729: Abcam, Cambridge, UK) were added into the samples. In each group, 80 μL of the samples was added into 96-well plate, followed by 50 μL of 5 mM p-nitrophenylphosphate (pNPP, Ab146203: Abcam, Cambridge, UK). Standard of pNPP was prepared at the same time by diluting 5 mM of pNPP with ALP buffer to obtain 1 mM of pNPP. Then, standard was placed into each well to produce pNPP standards of 0, 4, 8, 12, 16, and 20 nmol/well. The final volume in each well was adjusted to 120 μL by adding ALP assay buffer. Ten μL of ALP enzyme solution was added and incubated for 1 hour at ambient temperature in dark condition. After incubation, 20 μL of stop solution was added. The absorbance was measured at 405 nm using microplate reader. The optical density (OD) data of the samples were obtained by comparing with standard curve following this formula:

B =Amount of *p* NP in sample that obtained from standard curve
ΔT = Reaction time (1 h)
V = Volume of original sample that added to the reaction (adjust to 80 μL)
D = Sample dilution factor

To obtain the relative ALP activity, the total ALP activity calculated from this formula was normalized in a proportion of total protein calculated from Bradford assay.



The appearance of mineralization in osteoblasts was studied by alizarin red staining. Briefly, 1% of alizarin red S solution (Sigma-Aldrich, St. Louis, Missouri, US) was dissolved in distilled water and adjusted to the pH of 4.2, then filtered through a 0.22 µm syringe filter (Pall corporation, Port Washington, New York, US). After 1, 3, 6, 9, and 12 days of incubation, old medium was removed. The cells were washed three times with PBS, fixed with cold absolute methanol for 5 minutes, and then incubated for 30 minutes at room temperature in the dark condition. After incubation, the excess dye was carefully washed with distilled water. Finally, images of TNF-α-treated osteoblasts cultured without or with PDLSCs-CM at different concentrations were captured under an optical microscope.

### Statistical Analysis

The distribution of all data was examined with Shapiro–Wilk test. Data were expressed as median (P25, P75). The differences in gene expression of TNF-α-challenged osteoblasts at 24 and 48 hours were analyzed with Mann–Whitney U test. The effect of PDLSCs-CM on gene expression and ALP activity among all experimental groups were analyzed with Kruskal–Wallis test. Then, Pairwise Comparison of Group was performed to compare the difference between groups. The statistical analysis was performed using SPSS software version 21.0 (IBM, Westchester County, New York, US). Statistical significance was considered with *p-* value less than 0.05 in all analyzes.

## Results

### Gene Expression of TNF-α-Challenged Osteoblasts

**Fig. 1:**
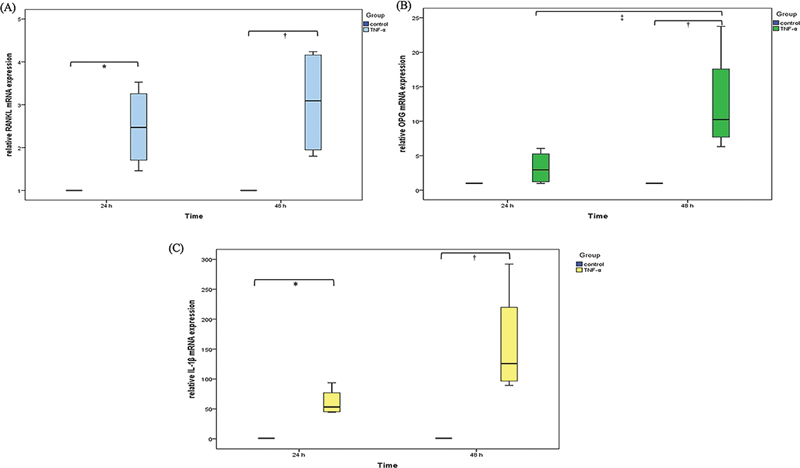
Expression of mRNA level in tumor necrosis factor-alpha (TNF-α)-treated osteoblasts and untreated control. ( **A** ) Receptor activator of NF-κB ligand (RANKL), ( **B** ) osteoprotegerin (OPG), and ( **C** ) interleukin-1β (IL-1β) ( *n* = 4, each). *Statistically significant differences between groups at 24 hours ( *p <* 0.05). ^†^ Statistically significant differences between groups at 48 hours ( *p <* 0.05). ^‡^ Statistically significant differences between 24 and 48 hours ( *p <* 0.05).

There was an increase in gene expression in TNF-α-challenged osteoblasts as time passed. Compared to the untreated control, TNF-α-challenged osteoblasts expressed significantly higher expression of RANKL at 24 and 48 hours ( Fig. 1A ), OPG at 48 hours ( Fig. 1B ), and IL-1β at 24 and 48 hours ( Fig. 1C ). When compared within group, OPG expression in TNF-α-challenged osteoblasts was significantly increased from 24 to 48 hours ( *p <* 0.05). ( Fig. 1B ).

### Gene Expression of TNF-α -Challenged Osteoblasts Cultured without or with PDLSCs-Derived CM

**Fig. 2:**
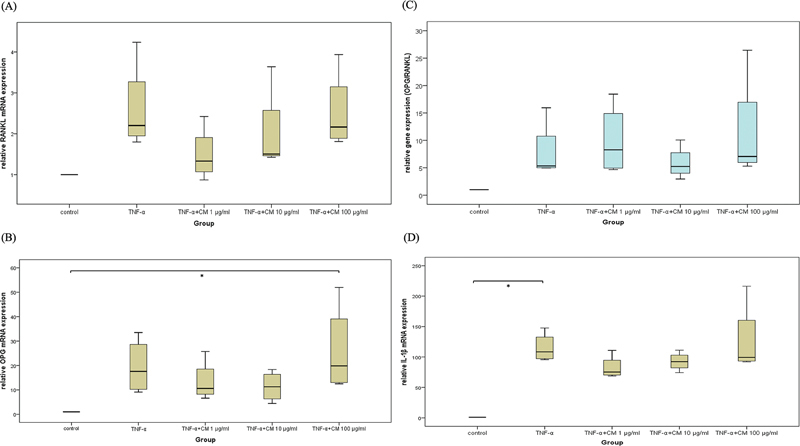
Expression of mRNA level in tumor necrosis factor alpha (TNF-α)-treated osteoblasts with periodontal ligament stem cells-derived conditioned medium (PDLSCs-CM) at 0, 1, 10, and 100 µg/mL. ( **A** ) receptor activator of NF-κB ligand (RANKL), ( **B** ) osteoprotegerin (OPG), ( **C** ) OPG/RANKL ratio, and ( **D** ) interleukin-1β (IL-1β; *n* = 4, each). * *p* < 0.05.

TNF-α increased the expression of RANKL in osteoblasts. The PDLSCs-CM at 1 μg/mL could downregulate the expression of TNF-α activated RANKL [1.33 (0.97, 2.17) vs 2.2 (1.87, 3.75)], which was comparable to a level of the control group [1.00 (1.00, 1.00)] ( *p* = 1.00). When PDLSCs-CM was increased to 10 and 100 μg/mL, the expression of RANKL was increased. However, the difference did not reach statistical significance ( Fig. 2A ).

Stimulation of OPG expression was seen in TNF-α-treated osteoblasts cultured without or with PDLSCs-CM ( Fig. 2B ). The PDLSCs-CM at 100 μg/mL significantly upregulated OPG expression compared to the control group ( *p* < 0.05) and closed to the osteoblasts treated with TNF-α. On the other hand, PDLSCs-CM at 1 μg/mL was the CM-treated group that least stimulated OPG expression as compared to the TNF-α group [10.58 (7.41, 22.11) vs 17.57 (9.66, 31.04)]. In addition, the PDLSCs-CM at 1 μg/mL tended to increase OPG/RANKL ratio compared to the TNF-α-challenged osteoblasts without PDLSCs-CM, but the difference did not reach statistical significance ( Fig. 2C ).

There was an elevation of IL-1β gene expression in TNF-α-challenged osteoblasts cultured without or with PDLSCs-CM. Between group comparison revealed a significant difference between TNF-α-treated osteoblasts without PDLSCs-CM and the control group ( *p <* 0.05). The group with 1 μg/mL PDLSCs-CM showed the most decreased expression of IL-1β among PDLSCs-CM group when compared to the TNF-α group without PDLSCs-CM [75.22 (69.64, 102.84) vs. 108.41 (96.51, 140.23)]. However, there was no significant difference between these two groups ( Fig. 2D ).

### Alkaline Phosphatase Activity of TNF-α-Challenged Osteoblasts

In the control group, ALP activity in osteoblasts significantly increased after 9 and 12 days of incubation compared to day 1 ( *p* = 0.008 and *p* = 0.001, respectively). When compared to the control group, ALP activity in TNF-α-treated osteoblasts decreased at day 3 until day 12, but significantly decreased at day 6 and 9 ( *p <* 0.05).

As shown in Table 2 , when the effect of PDLSCs-CM on ALP activity of TNF-α-challenged osteoblasts was evaluated, there was no significant difference between groups at day 1 and 3. A significant difference in ALP activity was found between groups at day 6, 9, and 12 ( *p* < 0.05). The PDLSCs-CM at 1 μg/mL showed a slightly elevated ALP activity [30.27 (14.72, 36.77)] compared to the TNF-α-challenged osteoblasts without PDLSCs-CM [18.16 (15.51, 40.88)] at day 12.

**Table 2:** Relative ALP activity in human osteoblasts of all experimental groups after 1, 3, 6, 9, and 12 days of incubation

Group	*n*	Relative ALP expression Median (P25, P75)										
		Day 1	*n*	Day 3	*n*	Day 6	*n*	Day 9	*n*	Day 12	*n*	*p-* Value
Control	5	11.56 (9.00, 22.15)	5	71.09 (42.16, 111.61)	5	140.69 (111.43, 185.55)	5	187.87 (161.03, 328.15) a	5	274.19 (224.20, 309.50) a	5	<0.001 ^†^
TNF-α	5	14.34 (12.10, 26.29)	5	17.26 (14.76, 36.48)	5	11.63 (8.47, 17.51) b	5	27.85 (13.98, 29.90) b	5	18.16 (15.51, 40.88)	5	0.141
TNF-α + CM 1 μg/mL	5	15.11 (14.08, 25.58)	5	10.52 7.94, 41.02)	5	11.18 (9.93, 17.50)	5	26.27 (21.44, 29.80)	5	30.27 (14.72, 36.77)	5	0.138
TNF-α + CM 10 μg/mL	5	15.36 (11.88, 23.67)	5	15.55 (13.32, 42.67)	5	12.34 (9.66, 20.61)	5	30.56 (15.29, 35.32)	5	12.98 (8.69, 17.29)	5	0.139
TNF-α + CM 100 μg/mL	5	17.59 (14.05, 22.39)	5	18.09 (13.44, 36.44)	5	16.01 (11.20, 20.34)	5	27.46 (15.00, 37.96)	5	17.71 (15.13, 34.09)	5	0.613
*p-* Value		0.639		0.057		0.014*		0.016*		0.005*		

Abbreviation: ALP, alkaline phosphatase; CM, conditioned medium; TNF-α, tumor necrosis factor-alpha.

*, *p <* 0.05; ^†^ , *p* < 0.001 ( *n* = 5, each).

a , Statistically significant difference when compared to the control group on day 1.

b , Statistically significant difference when compared to the control group in the same day.

### Alizarin Red Staining of TNF-α -Challenged Osteoblasts

**Fig. 3:**
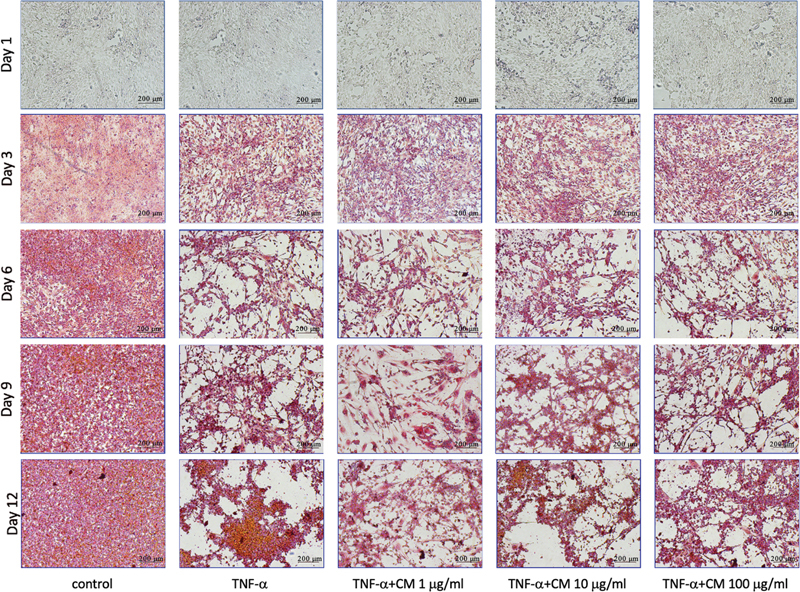
Late stage of osteoblast differentiation observed with alizarin red S staining at day 1, 3, 6, 9, and 12. CM, conditioned medium; TNF-α, tumor necrosis factor alpha.

At day 1, intracellular calcium formation observed as red deposits was not seen in all groups. The calcium deposits could be found on day 3. The amount of alizarin red S staining in the TNF-α-challenged groups cultured with PDLSCs-CM was comparable to that without PDLSCs-CM on the same incubation day ( Fig. 3 ).

## Discussion

In patients with periodontitis, TNF-α in gingival crevicular fluid was ranged from 0.10 to 700,000 pg/mL. 19 This cytokine has a paradoxical effect in inhibiting or activating osteoblastogenesis depending on its concentration and exposure time as well as the differentiation stage of the responding cells, that is, mediates early stage of osteogenic differentiation and suppresses osteoblastogenesis when MSCs are ready for the differentiation process. 2 TNF-α also influences osteoclast precursor differentiation and bone resorption activity through the induction of RANKL expression within osteogenic cells. 2 Recent studies found that 10 and 100 ng/mL of TNF-α could stimulate RANKL expression of osteoblasts within 24 hours 16 20 and 3 days, 21 respectively. Therefore, we designed the model mimicking bone loss in periodontitis by using TNF-α stimulated human osteoblasts and found that 50 ng/mL of TNF-α could significantly upregulate RANKL expression at 24 and 48 hours.

Regarding OPG, our study showed that TNF-α significantly upregulated OPG mRNA level after 48 hours of incubation. This might be due to the effect of TNF-α itself and the permissive incubation temperature used in this study (34°C). It was shown that the production of OPG by cultured osteoblasts increased with cell differentiation. 22 The hFOB 1.19 cells, which are human fetal osteoblastic cell line, are conditionally coded with a temperature-sensitive mutant of the SV40 large T antigen ( *ts-SV40LTA* ) gene. When the cells were cultured at permissive temperature (33.5°C), they proliferated rapidly. On the other hand, they demonstrated less or no proliferation and instead spontaneously differentiated into mature osteoblastic phenotype when cultured at restrictive temperature (39.5°C). 23 Thus, our finding could be, in part, explained by changing of the incubation temperature and thus, increasing osteoblast differentiation and expression of OPG.

To our knowledge, this study was the first to evaluate the effect of PDLSCs-CM on gene expression of TNF-α-challenged osteoblasts. The concentrations of PDLSCs-CM were selected based on the previous study in a mouse preosteoblasts model. 24 In that study, they investigated the protein concentration of RANKL and OPG in MC3T3-E1 osteoblasts treated with soybean extract. 24 They found that 1 and 100 μg/mL of soybean extract significantly increased the protein level of OPG in a dose-dependent manner. On the other hand, RANKL was significantly attenuated at 1 μg/mL, but slightly increased at 100 μg/mL of soybean extract. 24 Thus, the concentration of PDLSCs-CM at 1, 10, and 100 μg/mL was used in this experiment.

The results of this study indicated that PDLSCs-CM did not significantly alter the expression of genes related to bone homeostasis in TNF- α-challenged osteoblasts. The PDLSCs-CM at 1 and 10 μg/mL tended to downregulate OPG mRNA level of TNF-α-challenged osteoblasts compared to the group without PDLSCs-CM, although the difference did not reach statistical significance. As TNF-α itself significantly upregulated OPG mRNA expression of osteoblasts, it seemed that PDLSCs-CM at low concentration could attenuate the effect of TNF-α on the expression of OPG.

Besides the individual expression of OPG and RANKL, OPG/RANKL ratio is recommended to use as a major determinant of bone homeostasis since the process is regulated by RANK/RANKL/OPG system. In this study, the PDLSCs-CM at 1 μg/mL tended to increase OPG/RANKL ratio compared to the TNF-α-challenged osteoblasts without PDLSCs-CM. It was found that, in human periodontitis biopsies, RANKL mRNA expression levels were increased, while OPG expression levels were decreased, thus reducing the OPG/RANKL ratio. 25 Therefore, PDLSCs-CM at 1 μg/mL may demonstrate the benefits in reducing bone destruction as indicated by an increased OPG/RANKL ratio.

IL-1β plays a role in bone resorption by inducing formation of new osteoclasts from bone marrow precursors and activating osteoclasts to resorb bone through RANKL production by osteoblasts. 26 When osteoblasts were stimulated under pathological condition, IL-1β were significantly increased in 24 h. 27 Previous studies reported that CM from hPDLSCs decreased mRNA expression of IL-1β. 13 28 Similarly, the result from this study indicated that PDLSCs-CM at 1 μg/mL downregulated mRNA expression of IL-1β.

In this study, the effect of PDLSCs-CM on gene expression of OPG was different from that of RANKL and IL-1β. This could be explained by the different signaling pathways since TNF-α was signaled via the p38 MAPK pathway to mediate RANKL and IL-1 gene expression in murine marrow stromal cells and human mesenchymal stem cells (hMSCs), 29 or RANKL expression in osteocytes, 21 whereas Wnt pathway played a role in the mRNA expression of OPG in osteoblasts. 30

The PDLSCs-CM contained various cytokines that could be grouped into growth factors, proinflammatory and anti-inflammatory cytokines, and angiogenesis-related factors. 11 28 Several secretory proteins in PDLSCs-CM have been reported to exhibit immunomodulatory actions. 31 They can reduce the expression of IL-1β and TNF-α. 32 33 Previous study found that the differences in culture medium and supplements, culture duration and condition, as well as different passage and number of cells yielded the different level of cytokine in CM. 11 Therefore, this might be the reason why the concentration of CM affected the level of gene expression.

ALP is an enzyme involved in matrix maturation of early-stage bone formation. 34 In physiologic condition, ALP activity continued to increase in hFOB cells after incubation at 37°C for 3 days and reached a peak at 6 days, then continued to decline till day 12. 35 In contrast, our study observed an increased ALP activity in osteoblasts after 3 days of incubation and continued to increase for another 12 days. In terms of concentration, TNF-α at less than 1 ng/mL promoted osteogenic differentiation by upregulating ALP activity, while at higher concentrations of TNF-α (10 and 100 ng/mL), ALP activity reduced to a level less than the control after 48 hours of incubation. 36 37 In addition, TNF-α could inhibit intracellular calcium formation 38 and induce apoptosis of osteocytes. 39 Consistent to our findings, it was shown that osteoblasts treated with 50 ng/mL of TNF-α had lower ALP activity at day 3 and decreased in mineralization after 6 days compared to the control.

The effect of PDLSCs-CM at 0, 1, 10, and 100 μg/mL on ALP activity of TNF-α-challenged osteoblasts in our study showed no difference between groups. Only 1 μg/mL of PDLSCs-CM slightly increased ALP activity at day 12 when compared with TNF-α-treated group, but this different did not reach statistical significance. This might be due to the effect of TNF-α that could induce apoptosis of the cells since the group with 1 μg/mL PDLSCs-CM had more vital cells of hFOBs, while other groups showed an obvious decrease in the cell number after day 6 (data not shown).

In this study, a small sample size was a limitation. The PDLSCs-CM at 1 μg/mL resulted in an increased OPG/RANKL ratio of TNF-α-challenged osteoblasts, though not significant, it may be a new approach for the treatment of alveolar bone resorption. To prove this, larger sample size will be required in a further study. In addition, the components in PDLSCs-CM and the pathways involved in the effect of PDLSCs-CM on gene expression and ALP activity of osteoblasts should be explored.

## Conclusion

TNF-α mediated gene expression related to bone homeostasis including RANKL, OPG, and IL-1β, and diminished ALP activity in human osteoblasts. The PDLSCs-CM at 1 μg/mL tended to downregulate RANKL, OPG, and IL-1β gene expression of TNF-α-challenged osteoblasts compared to the TNF-α-challenged osteoblasts without PDLSCs-CM. Meanwhile, the PDLSCs-CM did not improve ALP activity of TNF-α-treated osteoblasts.

## References

[1] Working Group 2 of Seventh European Workshop on Periodontology Kinane D F Preshaw P M Loos B G Host-response: understanding the cellular and molecular mechanisms of host-microbial interactions–consensus of the Seventh European Workshop on Periodontology J Clin Periodontol 2011 38 11 44 48 21323703 10.1111/j.1600-051X.2010.01682.x

[2] Osta B Benedetti G Miossec P Classical and paradoxical effects of TNF-α on bone homeostasis Front Immunol 2014 5 48 24592264 10.3389/fimmu.2014.00048 PMC3923157

[3] Taubman M A Valverde P Han X Kawai T Immune response: the key to bone resorption in periodontal disease J Periodontol 2005 76(11, Suppl):2033 2041 10.1902/jop.2005.76.11-S.2033 16277573

[4] Karring T Nyman S Lindhe J Healing following implantation of periodontitis affected roots into bone tissue J Clin Periodontol 1980 7 02 96 105 6929795 10.1111/j.1600-051x.1980.tb01952.x

[5] Nyman S Karring T Lindhe J Plantén S Healing following implantation of periodontitis-affected roots into gingival connective tissue J Clin Periodontol 1980 7 05 394 401 6936413 10.1111/j.1600-051x.1980.tb02012.x

[6] Huang G T Gronthos S Shi S Mesenchymal stem cells derived from dental tissues vs. those from other sources: their biology and role in regenerative medicine J Dent Res 2009 88 09 792 806 19767575 10.1177/0022034509340867 PMC2830488

[7] Bartold M Gronthos S Haynes D Ivanovski S Mesenchymal stem cells and biologic factors leading to bone formation J Clin Periodontol 2019 46 21 12 32 10.1111/jcpe.13053 30624807

[8] Seo B M Miura M Gronthos S Investigation of multipotent postnatal stem cells from human periodontal ligament Lancet 2004 364(9429):149 155 15246727 10.1016/S0140-6736(04)16627-0

[9] Kinnaird T Stabile E Burnett M S Local delivery of marrow-derived stromal cells augments collateral perfusion through paracrine mechanisms Circulation 2004 109 12 1543 1549 15023891 10.1161/01.CIR.0000124062.31102.57

[10] Wada N Menicanin D Shi S Bartold P M Gronthos S Immunomodulatory properties of human periodontal ligament stem cells J Cell Physiol 2009 219 03 667 676 19160415 10.1002/jcp.21710

[11] Pawitan J A Prospect of stem cell conditioned medium in regenerative medicine BioMed Res Int 2014 2014 965849 25530971 10.1155/2014/965849 PMC4229962

[12] Yao S He H Gutierrez D L Expression of bone morphogenetic protein-6 in dental follicle stem cells and its effect on osteogenic differentiation Cells Tissues Organs 2013 198 06 438 447 24732882 10.1159/000360275 PMC4066880

[13] Huang C Y Vesvoranan O Yin X Anti-inflammatory effects of conditioned medium of periodontal ligament-derived stem cells on chondrocytes, synoviocytes, and meniscus cells Stem Cells Dev 2021 30 10 537 547 33757298 10.1089/scd.2021.0010

[14] Ballerini P Diomede F Petragnani N Conditioned medium from relapsing-remitting multiple sclerosis patients reduces the expression and release of inflammatory cytokines induced by LPS-gingivalis in THP-1 and MO3.13 cell lines Cytokine 2017 96 261 272 28511117 10.1016/j.cyto.2017.04.022

[15] Seubbuk S Sritanaudomchai H Kasetsuwan J Surarit R High glucose promotes the osteogenic differentiation capability of human periodontal ligament fibroblasts Mol Med Rep 2017 15 05 2788 2794 28447734 10.3892/mmr.2017.6333

[16] Zheng J Chen S Albiero M L Diabetes activates periodontal ligament fibroblasts via NF-κB in vivo J Dent Res 2018 97 05 580 588 29439598 10.1177/0022034518755697 PMC5958371

[17] Yeom J Ma S Lim Y H Probiotic *Propionibacterium freudenreichii* MJ2 enhances osteoblast differentiation and mineralization by increasing the OPG/RANKL ratio Microorganisms 2021 9 04 673 33805153 10.3390/microorganisms9040673 PMC8064112

[18] Eslaminejad M B Vahabi S Shariati M Nazarian H In vitro growth and characterization of stem cells from human dental pulp of deciduous versus permanent teeth J Dent (Tehran) 2010 7 04 185 195 21998794 PMC3184765

[19] Madureira D F Lucas De Abreu Lima I Costa G C Lages E MB Martins C C Aparecida Da Silva T Tumor necrosis factor-alpha in gingival crevicular fluid as a diagnostic marker for periodontal diseases: a systematic review J Evid Based Dent Pract 2018 18 04 315 331 30514445 10.1016/j.jebdp.2018.04.001

[20] Pacios S Xiao W Mattos M Osteoblast lineage cells play an essential role in periodontal bone loss through activation of nuclear factor-kappa B Sci Rep 2015 5 16694 26666569 10.1038/srep16694 PMC4678879

[21] Marahleh A Kitaura H Ohori F TNF-α directly enhances osteocyte RANKL expression and promotes osteoclast formation Front Immunol 2019 10 2925 31921183 10.3389/fimmu.2019.02925 PMC6923682

[22] Kearns A E Khosla S Kostenuik P J Receptor activator of nuclear factor kappaB ligand and osteoprotegerin regulation of bone remodeling in health and disease Endocr Rev 2008 29 02 155 192 18057140 10.1210/er.2007-0014 PMC2528846

[23] Yen M L Chien C C Chiu I M Multilineage differentiation and characterization of the human fetal osteoblastic 1.19 cell line: a possible in vitro model of human mesenchymal progenitors Stem Cells 2007 25 01 125 131 17204605 10.1634/stemcells.2006-0295

[24] Park K Ju W C Yeo J H Increased OPG/RANKL ratio in the conditioned medium of soybean-treated osteoblasts suppresses RANKL-induced osteoclast differentiation Int J Mol Med 2014 33 01 178 184 24248634 10.3892/ijmm.2013.1557

[25] Wara-aswapati N Surarit R Chayasadom A Boch J A Pitiphat W RANKL upregulation associated with periodontitis and Porphyromonas gingivalis J Periodontol 2007 78 06 1062 1069 17539720 10.1902/jop.2007.060398

[26] Hienz S A Paliwal S Ivanovski S Mechanisms of bone resorption in periodontitis J Immunol Res 2015 2015 615486 26065002 10.1155/2015/615486 PMC4433701

[27] García-López S Villanueva R Meikle M C Alterations in the synthesis of IL-1β, TNF-α, IL-6, and their downstream targets RANKL and OPG by mouse calvarial osteoblasts in vitro: inhibition of bone resorption by cyclic mechanical strain Front Endocrinol (Lausanne) 2013 4 160 24194731 10.3389/fendo.2013.00160 PMC3809383

[28] Nagata M Iwasaki K Akazawa K Conditioned medium from periodontal ligament stem cells enhances periodontal regeneration Tissue Eng Part A 2017 23(9-10):367 377 28027709 10.1089/ten.tea.2016.0274 PMC5444511

[29] Wei S Kitaura H Zhou P Ross F P Teitelbaum S L IL-1 mediates TNF-induced osteoclastogenesis J Clin Invest 2005 115 02 282 290 15668736 10.1172/JCI23394 PMC544608

[30] Glass D A II Bialek P Ahn J D Canonical Wnt signaling in differentiated osteoblasts controls osteoclast differentiation Dev Cell 2005 8 05 751 764 15866165 10.1016/j.devcel.2005.02.017

[31] Laplante P Brillant-Marquis F Brissette M J MFG-E8 reprogramming of macrophages promotes wound healing by increased bFGF production and fibroblast functions J Invest Dermatol 2017 137 09 2005 2013 28526301 10.1016/j.jid.2017.04.030

[32] Albus E Sinningen K Winzer M Milk fat globule-epidermal growth factor 8 (MFG-E8) is a novel anti-inflammatory factor in rheumatoid arthritis in mice and humans J Bone Miner Res 2016 31 03 596 605 26391522 10.1002/jbmr.2721 PMC6999704

[33] Deroide N Li X Lerouet D MFGE8 inhibits inflammasome-induced IL-1β production and limits postischemic cerebral injury J Clin Invest 2013 123 03 1176 1181 23454767 10.1172/JCI65167 PMC3582131

[34] Infante A Rodríguez C I Osteogenesis and aging: lessons from mesenchymal stem cells Stem Cell Res Ther 2018 9 01 244 30257716 10.1186/s13287-018-0995-x PMC6158877

[35] Donahue H J Li Z Zhou Z Yellowley C E Differentiation of human fetal osteoblastic cells and gap junctional intercellular communication Am J Physiol Cell Physiol 2000 278 02 C315 C322 10666026 10.1152/ajpcell.2000.278.2.C315

[36] Huang H Zhao N Xu X Dose-specific effects of tumor necrosis factor alpha on osteogenic differentiation of mesenchymal stem cells Cell Prolif 2011 44 05 420 427 21951285 10.1111/j.1365-2184.2011.00769.x PMC6495272

[37] Glass G E Chan J K Freidin A Feldmann M Horwood N J Nanchahal J TNF-alpha promotes fracture repair by augmenting the recruitment and differentiation of muscle-derived stromal cells Proc Natl Acad Sci U S A 2011 108 04 1585 1590 21209334 10.1073/pnas.1018501108 PMC3029750

[38] Bakker A D Silva V C Krishnan R Tumor necrosis factor alpha and interleukin-1beta modulate calcium and nitric oxide signaling in mechanically stimulated osteocytes Arthritis Rheum 2009 60 11 3336 3345 19877030 10.1002/art.24920

[39] Tan S D Kuijpers-Jagtman A M Semeins C M Fluid shear stress inhibits TNFalpha-induced osteocyte apoptosis J Dent Res 2006 85 10 905 909 16998129 10.1177/154405910608501006

